# Manganese nanoparticles control the gene regulations against multiple stresses in *Pangasianodon hypophthalmus*

**DOI:** 10.1038/s41598-023-43084-z

**Published:** 2023-09-23

**Authors:** Neeraj Kumar, Supriya Tukaram Thorat, Ajay Kumar Singh, Sanjivkumar Angadrao Kochewad, Kotha Sammi Reddy

**Affiliations:** ICAR-National Institute of Abiotic Stress Management, Baramati, Pune, 413115 India

**Keywords:** Zoology, Ichthyology, Gene regulation in immune cells, Innate immunity

## Abstract

Ammonia and arsenic pollution, along with the impact of climate change, represent critical factors influencing both the quantity and quality of aquaculture production. Recent developments have underscored the significance of these issues, as they not only disrupt aquatic ecosystems but also have far reaching consequences for human health. To addressed above challenges, an experiment was conducted to delineate the potential of manganese nanoparticles (Mn-NPs) to mitigate arsenic and ammonia pollution as well as high temperature stress in *Pangasianodon hypophthalmus*. The fish were exposed to different combination of arsenic and ammonia pollution as well as high temperature stress, while simultaneously incorporating diets enriched with Mn-NPs. The inclusion of Mn-NPs at 3 mg kg^−1^ in the diet led to a noteworthy downregulation of cortisol and HSP 70 gene expression, indicating their potential in mitigating stress responses. Furthermore, immune related gene expressions were markedly altered in response to the stressors but demonstrated improvement with the Mn-NPs diet. Interestingly, the expression of inducible nitric oxide synthase (iNOS), caspase (CAS), metallothionine (MT) and cytochrome P450 (CYP450) genes expression were prominently upregulated, signifying a stress response. Whereas, Mn-NPs at 3 mg kg^−1^ diet was significantly downregulated theses gene expression and reduces the stress. In addition to stress-related genes, we evaluated the growth-related gene expressions such as growth hormone (GH), growth hormone regulator 1 (GHR1 and GHRβ), Insulin like growth factor (IGF1 and IGF2) were significantly upregulated whereas, myostatin and somatostatin were downregulated upon the supplementation of dietary Mn-NPs with or without stressors in fish. The gene expression of DNA damage inducible protein and DNA damage in response to head DNA % and tail DNA % was protected by Mn-NPs diets. Furthermore, Mn-NPs demonstrated a capacity to enhance the detoxification of arsenic in different fish tissues, resulting in reduced bioaccumulation of arsenic in muscle and other tissues. This finding highlights Mn-NPs as a potential solution for addressing bioaccumulation associated risks. Our study aimed to comprehensively examined the role of dietary Mn-NPs in mitigating the multiple stressors using gene regulation mechanisms, with enhancing the productive performance of* P. hypophthalmus*.

## Introduction

Aquatic organisms have become increasingly vulnerable due to recent climate change and era of pollution. The abrupt shifts in temperature, degradation of water quality, and the pervasive influence of pollution have collectively contributed to the degradation of the aquatic environment and its inhabitants. Over the past few decades, the climate has exhibited uncertain behaviour, marked by fluctuation in heat and cold wave, drought and flooding and elevated frequency of intense rainfall. These changes have had a profound impact on the quality and sustainability of aquatic life, particularly fish. Climate change would be more severe with pollution and degraded water qualities, which affect the aquatic organism and their existence. According to the Intergovernmental Panel on Climate Change (IPCC)^1^, if current trends persist, global warming could increase by 1.5 °C between 2030–2052. One pressing concern in this context is the widespread contamination of arsenic (As), a potent toxic element. While its impact spans the globe, it is particularly pronounced in Southeast Asian countries. Disturbingly, the problem extends further, with over 100 countries grappling with arsenic-contaminated groundwater^2–4^. The consequences of arsenic contamination are dire, especially in Bangladesh, where more than 43,000 individuals succumb to its effects annually. The spectre of arsenic toxicity looms over more than 230 million people, with a staggering 180 million of them residing in various Asian nations^4,5^.

The aquaculture sector is grappling with the impact of undesirable abiotic and biotic factors that exert a significant influence productivity, production and product quality, ultimately affecting the health status of consumers. Particularly, factors such as ammonia and temperature play a pivotal role in limiting factors within the realms of aquaculture and fisheries. Ammonia in un-ionized form (NH_3_) is highly toxic to fish health and induces stress in culture environment^6^. The toxic substance not only induce stress but also disrupt the equilibrium of the aquatic habitat. Ammonia is produced due to decomposition of nitrogen-containing organic matter viz. fish, animal and plant carcasses, fish excretions, residues, and secretions^7^. Ammonia toxicity could be altered the growth performance, immune suppression, oxidative stress, tissue erosion, physiological disturbance, and disruption in the cytokines and NFkB signaling pathway. resulting into death of aquatic animals^8,9^. The delicate balance of temperature influences both the activity of aquatic organisms and the chemical reactions governing the aquatic environment. Fluctuations in temperature can set off a chain reaction of effects that reverberate through the ecosystem, profoundly affecting the success and viability of aquaculture.

Aquatic animals, being poikilothermic, exhibits a remarkable sensitivity to even minor shifts in water temperature, promoting comprehensive alterations in their physiological processes. The fish need more energy to maintained their body temperature similar to surrounding temperature. In addition, the temperature also affects on metabolism, osmoregulation, reproduction and behavior^9,10^. The daily oscillations in air temperature, the volume and rate of water flow, the dimensions of water bodies, the extent of mixing, and the influence of solar radiation collectively contribute to the mosaic of water temperature dynamics^11^.

Manganese nanoparticles (Mn-NPs) represent a micronutrient of profound significance in the realm of metabolic and biochemical processes^12^. This vital micronutrient assumes a multifaceted role, extending its influence to encompass protection against reactive oxygen species (ROS), immune-modulation, growth performance, blood coagulation, bone growth and functioning of nervous systems^13,14^. In the present investigation, we supplemented the Mn-NPs for alleviation of multiple stressors in fish due to certain important role in protection against stress. Mn-NPs also feature prominently involved in regulation of several biochemical reactions as integral part of metalloenzymes and activator of enzymes viz. glycosyltransferase, pyruvate carboxylase, alkaline phosphatase, glutamine synthetase, and especially mitochondrial manganese superoxide dismutase (Mn-SOD)^14^. A distinctive advantage of Mn-NPs over conventional manganese lies in their diminutive size and heightened biological reactivity. This characteristic augment their capacity to be absorbed and utilized within biological systems. This in turn leads to diminished excretion of this element into the environment and a more efficacious delivery of nutrients to fish, enhancing their overall well-being.

The stress responsive genes, encompassing apoptosis, cytokines, nuclear factor kappa B (NF-κB), immune genes, growth regulatory gene, and anti-oxidative gene regulators, hold pivotal significance in stress-induced scenarios. In a parallel vein, apoptosis emerges as a critical biological process recognised as programmed” cell death. This process bestows cellular functionality, regular cell repair, development of immune and hormone-related gene, and chemical cell death in all the organisms, including fish^15^. Similarly, the role of NF-kB is integral; it governs and modulates the transcription of genes associated with, inflammation, proliferation, the cell cycle, and cell death^16^. Considering that, it is a scientific duty to reduce the impact of abiotic and biotic stress in aquatic ecosystems using novel approaches such as nutrition to mitigate the impact.

*Pangasianodon hypophthalmus* characterised by its rapid growth and remarkable stress tolerance, emerges as a prime candidate for expanding the roster of aquaculture species. Its allure is further augmented by its sought-after medicinal attributes and delectable taste, making it a high-demand commodity^17–19^. Within the scope of our present investigation, we present a pioneering exploration into the mechanistic role of Mn-NPs in countering the adverse effects of arsenic and ammonia toxicity, as well as the challenges posed by elevated temperature stress in this fish species. The overarching objective of this research endeavour is twofold: to unravel the intricate mechanisms through which Mn-NPs mitigate stressors and to shed light on the identification and control of genes intricately involved in stress responses within *P. hypophthalmus*. In essence, this study not only seeks to enhance our understanding of stress mitigation using Mn-NPs but also aims to uncover the genetic underpinnings that drive stress responses in this specific fish species.

## Material and methods

### Ethics statement

In this study, we strictly adhered to the ARRIVE guideline (Animal Research: Reporting of In Vivo Experiments) to ensure rigorous reporting standards. The methodology and outcome measures of the study were approved by the Director, NIASM and Research Advisory Committee (RAC). Additionally, our experimental procedures were in full compliance with both International and National Guidelines for the ethical treatment and caring the animal during the experiment.

### Experimental animal and design

The fish were collected from the farm pond of ICAR-National Institute of Abiotic Stress Management. The average weight and size of the fish was recorded as 6.94 ± 0.78 and 5.87 ± 0.23 cm respectively. The fish were kept in rectangular plastic tank with capacity of 150 L each. Prior to the experiment, the fish were quarantine under 1% dip salt solution and potassium permanganate (KMnO_4_) solution. To conduct the experiment, a total of 12 treatment groups were designed in triplicate. These groups encompassed various stressor combinations, including control, arsenic (As), ammonia (NH_3_), arsenic and ammonia (As + NH_3_), ammonia and high temperature (NH_3_ + T), arsenic, ammonia and high temperature (As + NH_3_ + T), manganese nanoparticles fed group at 2, 3 and 4 mg kg^−1^ diet with or without stressors. Each tank was stocked with fifteen fish amounting to a total of five hundred forty fish utilized in the study. Details treatment information is presented in the Table 1. The fish were fed Mn-NPs diets twice a day at 9:00 AM and 5:00 PM. Routine maintenance included removal of uneaten feeds and faecal matter through siphoning on a day basis. The water quality parameters were periodically analysed using APHA method^20^, and the parameters consistently fell within suitable range for the species being cultured^21^. Water replacement involving 2/3rd of the tank volume was carried out every alternate day. The arsenite (NaAsO_2_) and (NH_4_)_2_SO_4_ solution were maintained 100 mg L^−1^ of stock solutions for complete experiment. Ammonia toxicity was induced by adding ammonium sulfate ((NH4)_2_SO_4_) as a source of ammonia (NH_3_), and arsenic stress was induced by adding sodium arsenite (NaAsO_2_), both maintained at 1/10th of the LC_50_ values (2.0 mg L^−1^ for (NH_4_)_2_SO_4_ and 2.68 mg L^−1^ for arsenic)^9,22^. A temperature of 34 °C was upheld to induce high-temperature stress throughout the experiment. Aeration was provided via a compressed air pump. Four different Mn-NPs-containing pelleted diets were prepared, maintaining isocaloric (365 kcal/100 g) and isonitrogenous (35% crude protein) compositions. Feed ingredients included wheat flour, groundnut meal, soybean meal, and fish meal. Labile nutrients such as cod liver oil, lecithin, and vitamin C were added post-heating the feed ingredients. A manganese nanoparticles (Mn-NPs) free mineral mixture was manually prepared. Proximate analysis was conducted using AOAC^23^ methods, ether extract (EE) was determined through solvent extraction, crude protein via nitrogen content, and ash content through muffle furnace at 550 °C. Total carbohydrate content was calculated using the formula: 100—(CP% + EE% + Ash% + Moisture). Gross energy was determined following the Halver method^24^, as outlined in Table 2.

**Table 1:** Experimental design of present investigation.

S. No	Details of the treatments	Notation
1	Control	Ctr
2	Fed with control diet and exposure to arsenic	As
3	Fed with control diet and exposure to ammonia	NH_3_
4	Fed with control diet and concurrently exposure to arsenic and ammonia	As + NH_3_
5	Fed with control diet and concurrent exposure to ammonia and high temperature	NH_3_ + T
6	Fed with control diet and concurrent exposure to arsenic, ammonia and high temperature	As + NH_3_ + T
7	Fed with manganese nanoparticles at 2 mg kg^−1^ diet	Mn-NPs at 2 mg kg^−1^ diet
8	Fed with manganese nanoparticles at 3 mg kg^−1^ diet	Mn-NPs at 3 mg kg^−1^ diet
9	Fed with manganese nanoparticles at 4 mg kg^−1^ diet	Mn-NPs at 4 mg kg^−1^ diet
10	Fed with manganese nanoparticles at 2 mg kg^−1^ diet and concurrent exposure to arsenic, ammonia and high temperature	Mn-NPs at 2 mg kg^−1^ diet + As + NH_3_ + T
11	Fed with manganese nanoparticles at 3 mg kg^−1^ diet and concurrent exposure to arsenic, ammonia and high temperature	Mn-NPs at 3 mg kg^−1^ diet + As + NH_3_ + T
12	Fed with manganese nanoparticles at 4 mg kg^−1^ diet and concurrent exposure to arsenic, ammonia and high temperature and	Mn-NPs at 4 mg kg^−1^ diet + As + NH_3_ + T

**Table 2:** Ingredient composition and proximate analysis of experimental diets (% dry matter) of manganese nanoparticles (Mn-NPs), diet fed to *Pangasianodon hypophthalmus* during the experimental period of 105 days.

Feed ingredients	Mn-NPs-0 mg kg^−1^ diet	Mn-NPs-2 mg kg^−1^ diet	Mn-NPs-3 mg kg^−1^ diet	Mn-NPs-4 mg kg^−1^ diet
Soybean meal^a^	35.5	35.5	35.5	35.5
Fish meal^a^	25	25	25	25
Groundnut meal^a^	10	10	10	10
Wheat flour^a^	17.2	17.198	17.197	17.196
Sunflower oil^a^	4.5	4.5	4.5	4.5
Cod liver oil^a^	1.5	1.5	1.5	1.5
CMC^b^	2	2	2	2
Vitamin and mineral mix*	2	2	2	2
Vitamin C^c^	0.3	0.3	0.3	0.3
Lecithin^b^	2	2	2	2
Mn-NPs	0	0.002	0.003	0.004
Proximate composition of the diets				
Crude protein (CP)	35.34 ± 0.39	35.16 ± 0.08	35.43 ± 0.18	35.17 ± 0.02
Ether extract (EE)	8.23 ± 0.09	8.57 ± 0.22	8.72 ± 0.31	8.34 ± 0.10
Total carbohydrate (TC)	40.37 ± 0.36	40.89 ± 0.68	40.58 ± 0.41	40.13 ± 0.08
Organic matter (OM)	92.05 ± 0.08	92.26 ± 0.28	91.98 ± 0.07	91.81 ± 0.02
Dry matter (DM)	91.90 ± 0.13	92.36 ± 0.30	92.74 ± 0.04	91.83 ± 0.13
Digestible energy (DE)	363.47 ± 0.93	364.20 ± 1.95	365.03 ± 0.99	364.59 ± 0.85

^a^Procured from local market, ^b^Himedia Ltd, Himedia Ltd, ^c^SD Fine Chemicals Ltd., India.

*Manual prepared Vitamin mineral mixture; Composition of vitamin mineral mix (quantity/250 g starch powder): vitamin A 55,00,00 IU; vitamin D3 11,00,00 IU; vitamin B1:20 mg; vitamin E 75 mg; vitamin K 1,00 mg; vitamin B12 0.6 mcg; calcium pantothenate 2,50 mg; nicotinamide 1000 mg; pyridoxine: 100 mg; Zn 500 mg; I 1,00 mg; Fe 750 mg; Cu 200 mg; Co 45 mg; Ca 50 g; P 30 g; Se: 2 ppm.

Digestible energy (DE) (Kcal/100 g) = (% CP × 4) + (% EE × 9) + (TC × 4).

Data expressed as mean ± SE, n = 3.

### Manganese nanoparticles (Mn-NPs) synthesis using green approach

#### Preparation of fish tissue extract

The preparation of fish tissue extract followed our established procedure^25,26^ for synthesis of Mn-NPs. In essence, fish tissues were employed for the synthesis of Mn-NPs. The gill tissue was meticulously washed and dissected under aseptic conditions. Subsequently, it was segmented into smaller pieces and homogenized using the Omni Tissue Master Homogenizer (Kennesaw, GA). The resulting homogenate was then subjected to centrifugation at 5000–6000 rpm, yielding a supernatant. This supernatant was subsequently filtered using Whatman paper with a pore size of 0.45 μm, resulting in the isolation of the gill extract.

#### Preparation and characterization of manganese nanoparticles (Mn-NPs)

**Figure 1:**
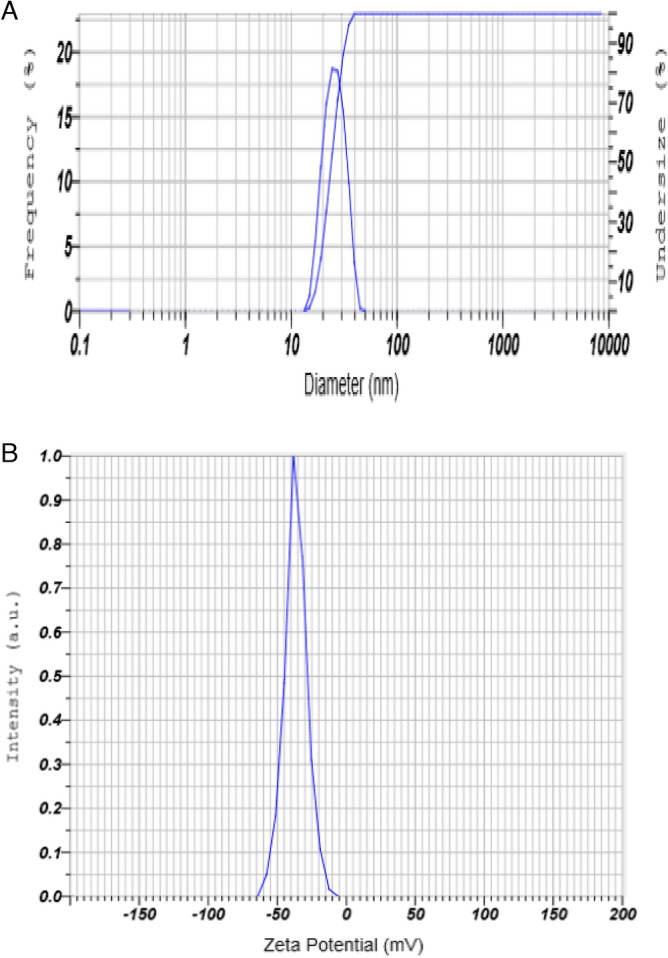
(**A**-**B**): Particle size (22 nm) and zeta potential (− 36.6 mV) of manganese nano particles (Mn-NPs).

**Figure 2:**
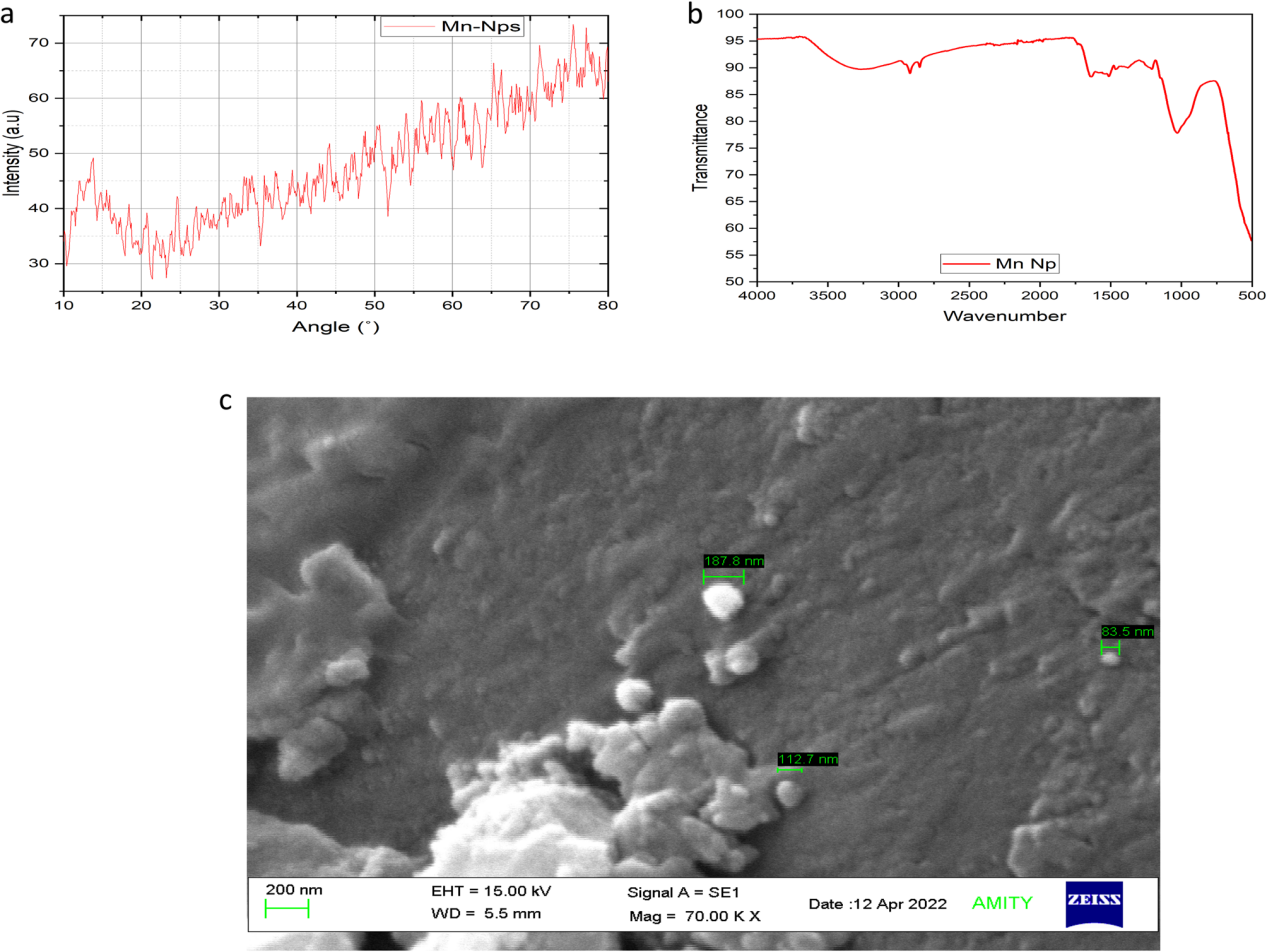
(**a**-**c**): Characterization of Mn-NPs using (**a**) X-Ray Diffraction (XRD), (**b**) Fourier transform infrared spectroscopy (FTIR) and (**c**) Scanning electron microscope.

The obtained gill extract was mixed with manganese acetate (1 mM) and maintained the solution to pH 6.8. Stirring was carried out for 1 h to facilitate metal reduction, resulting in a transformation of the solution to a reddish-brown. Following this, a curcumin solution (1 mM) was introduced into the mixture and allowed to react for 6 h at room temperature. The color of the solution transitioned to a permanent black, signifying the successful stabilization of the manganese nanoparticles (Mn-NPs). To isolate pure manganese nanoparticles, the final solution underwent a through washing process using de-ionised water, after which it was subjected to centrifugation at 5000–6000 rpm. The resultant product was then dried using an oven and stored at room temperature. For characterization, the absorption spectrum of the Mn-NPs was analysed at wavelength of 200–600 nm using spectrophotometer (Shimadzu, UV-1900i). This analysis revealed a distinct peak in the 410–430 nm range. Further characterization of the Mn-NPs was analysed by Nano Size analyser (Particle Analyser, Litesizer 500, Anton Paar, Austria) for size and zeta potential. The size and zeta potential of Mn-NPs was determining as 22 nm and − 36.6 mV respectively (Fig. 1). The Mn-NPs was also characterised by Fourier transform infrared spectroscopy (FTIR), X-Ray diffraction (XRD), and scanning electron microscope (Fig. 2).

### RNA isolation and quantification

For the isolation of total RNA from the liver tissue of *P. hypophthalmus*, the TRIzol method was employed. Liquid nitrogen was used for homogenisation of liver tissue. Subsequently, chloroform was added to the homogenated samples, and the mixture incubated for 5 min to facilitate phase separation. Following incubation, centrifugation was carried out to separate the RNA containing phase. The RNA pellet was then washed with 75% ethanol and allowed to air dried. Then RNA pellet was dissolved in nuclease free water and stored at − 80 °C for further use. To assess the quality of the extracted RNA, a 1% agarose gel was utilized. The Gel documentation system was used for RNA bands visualization (ChemiDocTM MP imaging system, Bio-Rad). Nano-drop spectrophotometer was used for RNA quantification (Thermo-scientific).

### cDNA synthesis and quantitative PCR

For cDNA synthesis, the RevertAid First strand cDNA synthesis kit (Thermo-scientific) was employed. The trace amounts of DNA were effectively removed by utilizing DNase I. The reaction mixture containing oligo dT primers (15 pmol) and RNA template (100 ng) mixed to a total volume of 12 µl. This reaction mixture was subjected to a 5 min incubation at 65 °C, followed by rapid cooling on ice. Subsequently, a solution containing 1.0 µl of reverse transcriptase enzyme, 2 µl dNTP Mix (10 mM) and 1 µl Ribo Lock RNase Inhibitor (20 U/µL) was then incubated at 60 °C for 42 min, followed by a final incubation at 70 °C for 5 min. The resulting synthesized cDNA was stored at − 20 °C for future use. To validate the successful cDNA synthesis, β-actin was used as a reference. Real-time PCR was performed using the SYBR Green method (Bio-Rad) in combination with gene-specific primers. The process involved initial denaturation at 95 °C for 10 min, followed by 39 cycles of cDNA amplification, with denaturation at 95 °C for 15 s and annealing at 60 °C for 1 minute^27^. The specific primer sequences utilized are detailed in Table 3.

**Table 3:** Details of primer for relative quantitative real-time PCR.

Gene	Primer sequence (5′ –3′)	Accession number
SOD	F-GTCCATCTTACCCGGTGCCC	XM_034299545.1
	R-CGAGAGAAGACCCGGAACGC	
CAT	F-AGCAGGCGGAGAAGTACCCA	XM_026919141.2
	R-GCTGCTCCACCTCAGCGAAA	
GPx	F- GTCACTGCAGGATGCAACAC	XM_026947312.2
	R- TTGGAATTCCGCTCATTGAT	
HSP 70	F- CTCCTCCTAAACCCCGAGTC	XM_026934573.2
	R- CCACCAGCACGTTAAACACA	
iNOS	F-ACACCACGGAGTGTGTTCGT	XM_026931613.2
	R-GGATGCATGGGACGTTGCTG	
DNA damage inducible protein	F-CACCTTCGCCCTCGAAGTCT	XM_026938137.2
	R-GCTCGGGTGAGGTCTCTCAG	
TNFα	F-TGGAGTTCTGCTTGCCGTGG	XM_026942329.2
	R-GCAGCCTTTGCAGTCTCGGA	
TLR	F: TCACCACGAACGAGACTTCATCC	XM_026916808.2
	R : GACAGCACGAAGACACAGCATC	
Ghr1	FTATTGGCTACAGCTCGCCGC	XM_034306157.1
	R-AATCACCCCGACTGTGCTGC	
Ghrb	F-TTGAGCTTTGGGACTCGGAC	XM_026942987.2
	R-CGTCGATCTTCTCGGTGAGG	
IGF-1X1	F-GCAACGGCACACAGACACGC	XM_034313382.2
	R-CAGACGTTCCCTCACCATCCTCT	
IGF-1X2	F-CGAGAGCAACGGCACACAGA	XM_034313383.2
	R-TTCTGATGGACCTCCTTACAAGATG	
IL	F- AGCAGGATCCATCAAAGTGG	XM_026918084.2
	R- GTGCTCCAGCTCTCTGGGTA	
Ig	F- GGCCAGTAATCGTACCTCCA	XM_026923540.2
	R- CTTCGTAAGGTCCCCACTGA	
MYST	F-GGGAAAGACCTGGCCGTGAC	XM_026910492.2
	R-TCGAGGCCGGATTCTCGTCT	
SMT	F- CTCTGGGTGGCAGAATGAAT	XM_026921272.2
	R- AACATGAAGAGAACGTTTTCCAG	
GH	F-CCCAGCAAGAACCTCGGCAA	GQ859589.1
	R-GCGGAGCCAGAGAGTCGTTC	
CYP P450	F-GATTCGGCATCCGTGCGTGC	NC_047599.1
	R-GATGTGGCTGGGACGAGCA	
MT	F-CACGGCTTTTCCTGTCCGCT	AF087935.1
	R-AACAGCGCCCCCAGGTGTC	
Cas 3a	F-CGGCATGAACCAGCGCAAC	NC_047622.1
	R-TCCACCGCACCATCTGTCCC	
Cas3b	F-AGCTTTCCGTGAGCTGGGCT	NC_047601.1
	R-TGGCTGACTTGCTGTGGTCCT	
Na^+^K^+^ATPase	F-AACTACAAGCCCACGTACCA	XM_026923907.3
	R-CTTGCCAGCCTTAAAGCCAA	
β-Actin	F-CAGCAAGCAGGAGTACGATG	XM_031749543.1
	R-TGTGTGGTGTGTGGTTGTTTTG	

SOD: Superoxide dismutase; CAT: Catalase; GPx: Glutathione peroxidase; HSP: Heat shock protein; iNOS: Nitric oxide synthase; TNFα: Tumor necrosis factor; TLR: Toll like receptor; Ghr: Growth hormone receptor; IL; Interleukin; Ig: Immunoglobulin; MYST: myostatin SMT; Somatostatin; CYP P450: Cytochrome P450; MT: Metallothionine; Cas 3a and 3b: caspase 3; GH: Growth hormone; IGF1 and 2: Insulin like growth factor.

### Growth performance

The growth performance was determined by evaluating following method. The sampling/weighing of the fish was observed by every 15 days up to 105 days.

$${\text{FCR }} = {\text{ Total dry feed intake }}\left( {\text{g}} \right)/{\text{Wet weight gain }}\left( {\text{g}} \right)$$

$${\text{SGR }} = { 1}00 \, \left( {{\text{ln FBW}} - {\text{ln IBW}}} \right)/{\text{ number of days}}$$

$${\text{Weight gain }}\left( \% \right) \, = {\text{ Final body weight }}\left( {{\text{FBW}}} \right) - {\text{Initial body weight }}\left( {{\text{IBW}}} \right)/{\text{Initial body weight }}\left( {{\text{IBW}}} \right) \, \times {1}00$$

$${\text{Relative feed intake}}, \, \left( {{\text{FI}}} \right) \, \left( {\% /{\text{d}}} \right) \, = { 1}00 \, \times \, \left( {{\text{TFI}}/{\rm I}{\text{BW}}} \right)$$

$${\text{PER}} = {\text{ Total wet weight gain }}\left( {\text{g}} \right)/{\text{crude protein intake }}\left( {\text{g}} \right)$$

$${\text{Thermal growth coefficient}}, \;\left( {{\text{TGC}}} \right) \; = \; \left( {{\text{FBW}}^{{1/3}} - {\text{IBW}}^{{1/3}} } \right) \; \times \; \left( {\Sigma {\text{D}}0} \right)^{{ - 1}} ,\;{\text{where}}\;\sum {\text{D0}}\;{\text{is}}\;{\text{the}}\;{\text{thermal}}\;{\text{sum (feeding}}\;{\text{days }} \times {\text{sum (average}}\;{\text{temperature, }}{^\circ }{\text{C}})$$

$${\text{Daily growth index}},{\text{ DGI }}\left( \% \right) \, = \, \left( {{\text{FBW}}^{{{1}/{3}}} {-}{\text{ IBW}}^{{{1}/{3}}} } \right)/{\text{days }} \times { 1}00$$

### Genes

The genes were investigated in liver tissues in this study viz. catalase (CAT), glutathione-s-transferase (GST), superoxide dismutase (SOD), nitric oxide synthase (iNOS), heat shock protein (HSP 70), Caspase 3a (CAS 3a and 3b), cytochrome P450 (CYP 450), tumor necrosis factor (TNFα), toll like receptor (TLR), metallothionine (MT), growth hormone receptor (Ghr1 and Ghrb), interleukin (IL), immunoglobulin (Ig), growth factor 1 and 2 (IGF1 and IGF 2) somatostatin (SMT), myostatin (MYST), insulin like and growth hormone (GH), studied for real-time quantification.

### Cortisol

ELISA kit was used for cortisol determination (Catalog no. 500360, Cayman Chemicals, USA) and followed the protocol provided by the kit. The final reading was obtained using ELISA plate reader (Biotek India Pvt. Ltd.).

### Manganese analysis from fish tissues and experimental water

Liver, muscle, gill, brain, and kidney tissues were collected to determine arsenic concentration. Whereas, Mn concentration was determined in the feed and fish muscle. The tissues samples and diets were undergoing processing within a microwave digestion system (Microwave Reaction System, Multiwave PRO, Anton Paar GmbH, Austria, Europe). The Inductively Coupled Plasma Mass Spectrometry (ICP-MS) technique (Agilent 7700 series, Agilent Technologies, USA) was employed for the analysis, in accordance with the methodology outlined by Kumar et al^28,29^.

### Alkaline single-cell gel electrophoresis (SCGE)/Comet assay

The alkaline single cell gel electrophoresis/comet assay was employed to determine DNA damage in kidney tissue using Ali et al^30^ with slight modification^31^. The kidney tissue (50 mg) was washed under double distilled water, then rinsed twice with chilled phosphate buffer saline (Ca^2+^ Mg^2+^ free), and subsequently placed in ice-cold homogenization buffer (20 mM EDTA; 1-X Hanks’ balanced salt solution; 10% dimethyl sulphoxide (DMSO), pH 7.0–7.5). From this tissue, a single cell suspension was obtained and subsequently centrifuged at 4 °C at and 3000 rpm for 5 min. The resulting cell pellets were re-suspended in phosphate saline buffer. To ensure cell viability, the trypan blue exclusion test method was applied^30^. The coating of slides and other procedural steps closely followed the aforementioned method. The slides, prepared for genotoxicity analysis, were observed under a fluorescent microscope (Leica Microsystems Ltd, DM 2000, Heerbrugg, Switzerland). The position of DNA damage was captured using Fluorescence microscope and analysed using Open comet software. For the quantification of DNA damage, the parameter chosen was percent tail DNA (expressed as % tail DNA = 100%—% head DNA), as determined by the software.

### Statistics

The data were analysed using Statistical Package for the Social Sciences (SPSS) version 16 software. Prior to analysis, normality and homogeneity of variance were assessed using the Shapiro–Wilk’s and Levene’s test and Shapiro–Wilk’s test, respectively. One way ANOVA (analysis of variance) using Duncan’s multiple range tests were applied in the present study. The data were analysed and significant at *p* < 0.05.

### Ethical approval

The Institute Research Advisory Committee (RAC) has approved the experimental procedures and this study compliance with Animal Research: Reporting of In Vivo Experiments (ARRIVE) guidelines.

### Consent to participate

All authors are aware and agree with this submission for publication.

## Results

### Characterization of Mn-NPs

The primary characterisation of Mn-NPs was performed using spectrophotometer at 200–600 nm. The characterization revealed a prominent peak at 410 nm. Subsequent analysis provided values for zeta potential (− 36 mV) and mean size (22 nm), as demonstrated in Fig. 1A,B. Further investigation of the particles involved a comprehensive examination through X-Ray Diffraction (XRD), Fourier Transform Infrared Spectroscopy (FTIR), and Scanning Electron Microscope (SEM), with the outcomes presented in Fig. 2A–C.

### Primary stress response with respect to multiple stresses and mitigate it with Mn-NPs

**Figure 3:**
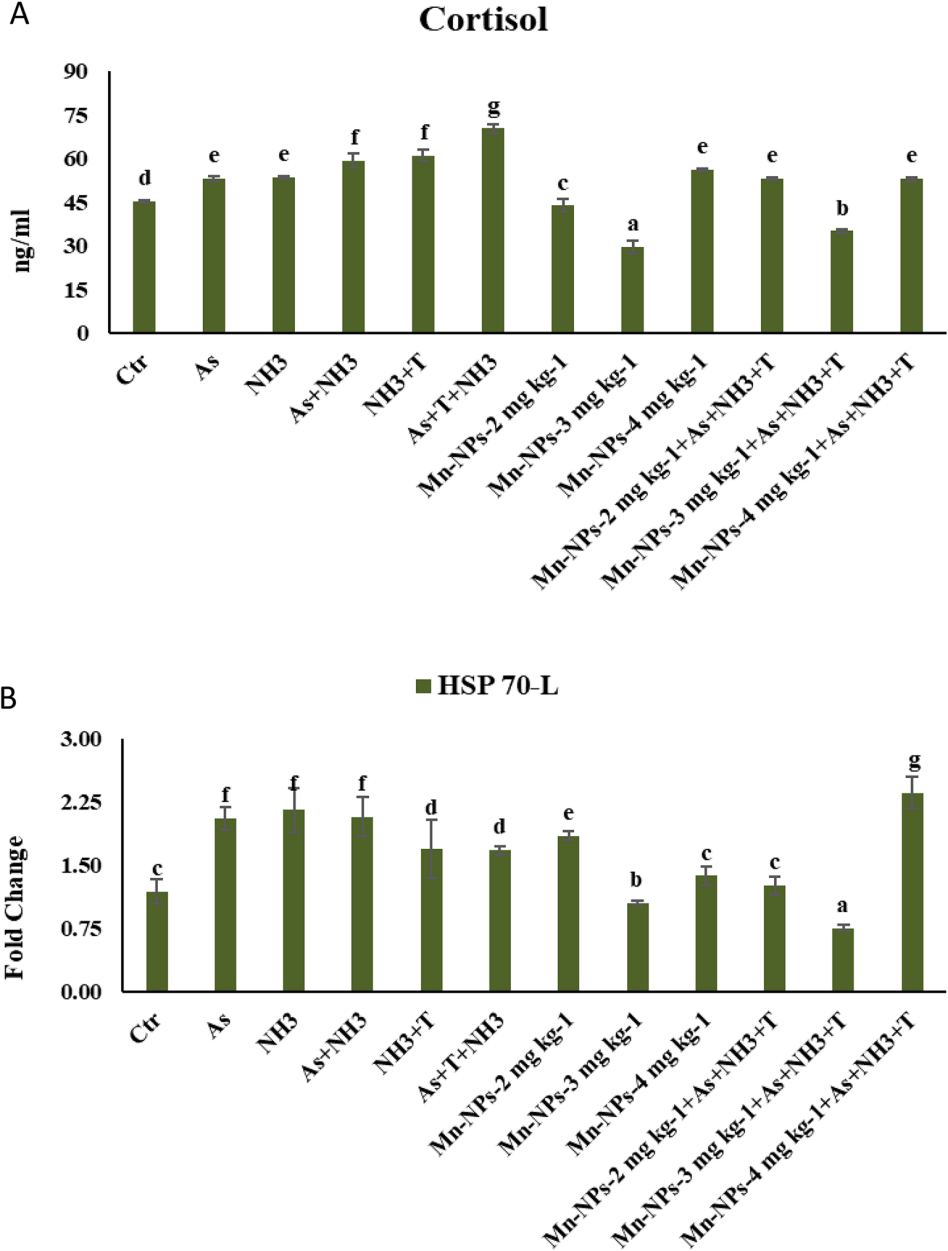
(**A**-**B**): Effect of Mn-NPs on mitigation of aresnic, ammonia and high temperature stress on cortisol and heat shock protein (HSP-70) in fish liver. Within endpoints and groups, bars with different superscripts differ significantly (**a**–**g**). Data expressed as Mean ± SE (n = 3). Cortisol: (*p* = 0.0070), liver HSP 70 (*p* = 0.0086).

The primary stress response, viz. cortisol was assessed in relation to arsenic, ammonia and high temperature stress. Additionally, different levels of Mn-NPs at 2, 3 and 4 mg kg^−1^ diet to were administered to *P. hypophthalmus*. Results indicated a substantial increase (*p* = 0.0070) in cortisol level under concurrent exposure to ammonia, arsenic and high temperature stress (As + NH_3_ + T). This was followed by elevated cortisol level in the As + NH_3_, NH_3_ + T and NH_3_, As group when compared to both control and Mn-NPs supplemented groups (Mn-NPs at 2 and 3 mg kg^−1^ diet). Furthermore, findings emerged with Mn-NPs at 3 mg kg^−1^ diet with or without stressors (As + NH_3_ + T), cortisol levels were significantly reduced. Similar cortisol reductions were observed with Mn-NPs at 2 mg kg^−1^ diet, but only when administered without stressors. These outcomes were in contrast to both control and stressor groups. In case of Mn-NPs at 4 mg kg^−1^ diet displayed notably higher cortisol levels when compared to the control and other Mn-NPs supplemented groups (Fig. 3A).

### Secondary stress response (HSP70) with respect to multiple stresses and mitigate it with Mn-NPs

The gene expression of heat shock protein 70 in liver (HSP 70-L) tissue of *P. hypophthalmus* subjected to ammonia, arsenic, and high temperature stress is presented in Fig. 3B. Notably, the expression of HSP 70 was downregulated (*p* = 0.0086) with group treated with Mn-NPs at 3 mg kg^−1^ diet, both with or without stressors (As + NH_3_ + T), when compared to control and other groups. Conversely, the gene expression of HSP 70 was significantly upregulated when Mn-NPs at 4 mg kg^−1^ diet fed with exposure to As + NH_3_ + T and followed As + NH_3_, As, NH_3_, NH_3_ + T and As + NH_3_ + T groups in comparison to control and other groups.

### Secondary stress response (DNA damage inducible protein and DNA damage, comet) with respect to multiple stresses and mitigate it with Mn-NPs

**Figure 4:**
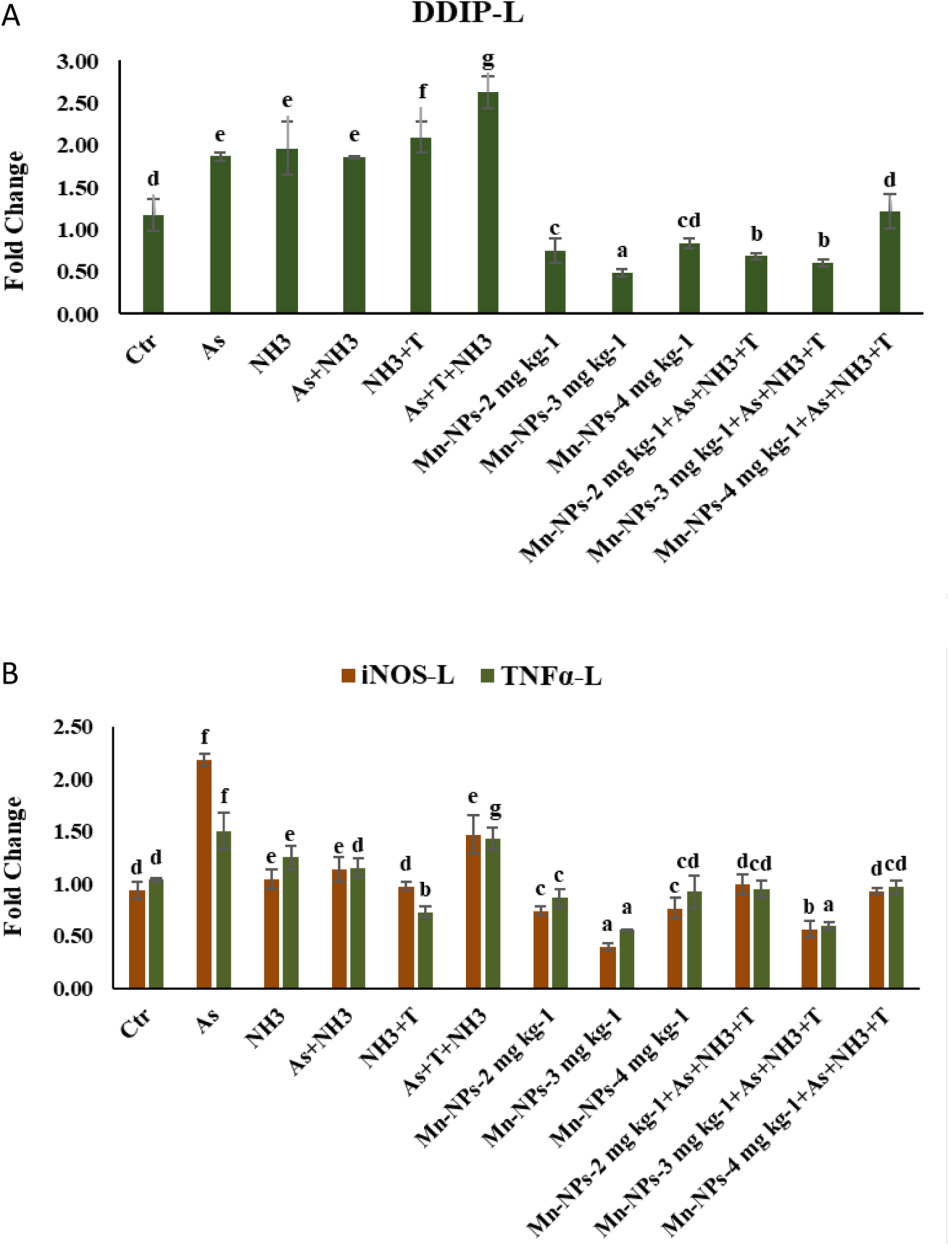
(**A**-**B**): Effect of Mn-NPs on mitigation of aresnic, ammonia and high temperature stress on DNA damage inducible protein (DDIP), inducible nitric oxide synthase (iNOS) and tumour necrosis factor (TNFα) in fish liver. Within endpoints and groups, bars with different superscripts differ significantly (**a**–**g**). Data expressed as Mean ± SE (n = 3). DDIP: (*p* = 0.0016), iNOS (*p* = 0.0035), TNFα (*p* = 0.0018).

The expression of DNA damage inducible protein (DDIP) gene exhibited a marked and significant upregulation (*p* = 0.0016) upon concurrent exposure to arsenic, ammonia and high temperature stress. This upregulation was further observed in the NH_3_ + T, As + NH_3_, NH_3_ and As group when compared to both control and the groups supplemented with Mn-NPs. Noteworthy, the supplementation of Mn-NPs at 3 mg kg^−1^ diet with or without stressors (As + NH_3_ + T), led to a substantial downregulation of DDIP expression when compared to control and stressors groups (Fig. 4A). Similarly, the DNA damage was observed in the kidney tissue in the form of tail DNA %, head DNA %, comet DNA, Head area, and comet length. The most significant tail DNA % was observed in the group exposed to As + NH_3_ + T (90.32%) followed by As + T (89.48%), NH_3_ + T (88.43) and As + NH_3_ (87.75%). In contrast, the control group exhibited the lowest tail DNA %, closely followed by the Mn-NPs at 2, 3 and 4 mg kg^−1^ diet without stressors (6.27, 6.53, 7.37%) respectively (Table 4).

**Table 4:** Mitigating role of Mn-NPs on genotoxicity (DNA damage) in kidney tissue of *P. hypophthalmus* reared under arsenic, ammonia and high temperature stress for 105 days.

Treatments	Comet Length	Comet DNA	Head Area	Head DNA	Head DNA (%)	Tail DNA	Tail DNA (%)
Control	35.0 ± 1.55	140,301 ± 23.98	942 ± 7.67	133,230 ± 26.67	94.96 ± 1.98	7071 ± 21.87	5.04 ± 0.44
As	30.0 ± 1.05	62,845 ± 15.34	156 ± 2.76	15,004 ± 27.09	23.87 ± 2.65	47,841 ± 20.71	76.13 ± 1.65
NH_3_	28.0 ± 0.67	46,116 ± 6.47	52 ± 1.45	4851 ± 11.45	10.52 ± 1.22	41,265 ± 31.87	89.48 ± 1.99
As + NH_3_	24.0 ± 0.93	48,760 ± 8.94	52 ± 1.21	5973 ± 16.32	12.25 ± 1.17	42,787 ± 16.69	87.75 ± 2.67
NH_3_ + T	48.0 ± 1.32	58,087 ± 9.27	51 ± 1.43	6723 ± 14.36	11.57 ± 1.05	51,364 ± 13.46	88.43 ± 3.61
As + T + NH_3_	23.0 ± 0.56	57,594 ± 11.54	52 ± 1.87	5573 ± 11.34	9.68 ± 0.56	52,021 ± 11.94	90.32 ± 4.87
Mn-NPs-2 mg kg^−1^	9.93 ± 0.15	23,179 ± 6.34	40 ± 1.81	3886 ± 11.34	6.27 ± 0.37	22,630 ± 22.76	6.27 ± 0.11
Mn-NPs-3 mg kg^−1^	22.0 ± 1.89	52,922 ± 7.93	377 ± 11.98	49,464 ± 25.87	93.47 ± 1.85	3458 ± 11.33	6.53 ± 0.21
Mn-NPs-4 mg kg^−1^	30.0 ± 1.48	58,541 ± 17.45	798 ± 13.76	54,224 ± 16.74	92.63 ± 1.25	4317 ± 17.56	7.37 ± 0.15
Mn-NPs-2 mg kg^−1^ + As + T + NH_3_	22.0 ± 1.01	48,292 ± 28.65	329 ± 11.71	40,212 ± 11.34	83.27 ± 1.67	8080 ± 9.32	16.73 ± 1.77
Mn-NPs-3 mg kg^−1^ + As + T + NH_3_	25.0 ± 1.36	49,287 ± 27.65	380 ± 7.56	42,443 ± 11.87	86.11 ± 1.15	6844 ± 13.23	13.89 ± 0.76
Mn-NPs-4 mg kg^−1^ + As + T + NH_3_	47.0 ± 1.76	71,698 ± 24.45	438 ± 8.36	41,374 ± 11.87	57.71 ± 1.09	30,324 ± 14.87	42.29 ± 0.65

Data expressed as Mean ± SE (n = 3).

### Secondary stress response (iNOS and TNFα) with respect to multiple stresses and mitigate it with Mn-NPs

The expression of inducible nitric oxide synthase (iNOS) gene in liver tissue exhibited a notable and significant upregulation (*p* = 0.0035) upon exposure to arsenic alone followed by As + NH_3_ + T, NH_3_ + T, As + NH_3_, and NH_3_ groups compared to control and Mn-NPs supplemented groups. Conversely, the iNOS gene expression was downregulated by Mn-NPs at 3 mg kg^−1^ diet with or without stressors followed by Mn-NPs at 2 mg kg^−1^ diet in comparison to control and stressors groups. Similarly, the gene expression of TNFα demonstrated a significantly upregulation (*p* = 0.0018) in As + NH_3_ + T and arsenic exposure group, followed by NH_3_ and As + NH_3_ groups. In contrast, Mn-NPs at 3 mg kg^−1^ diet led to a noticeable downregulation in comparison to the control and other groups (Fig. 4B).

### Secondary stress response (TLR, IL1β and Ig) with respect to multiple stresses and mitigate it with Mn-NPs

**Figure 5:**
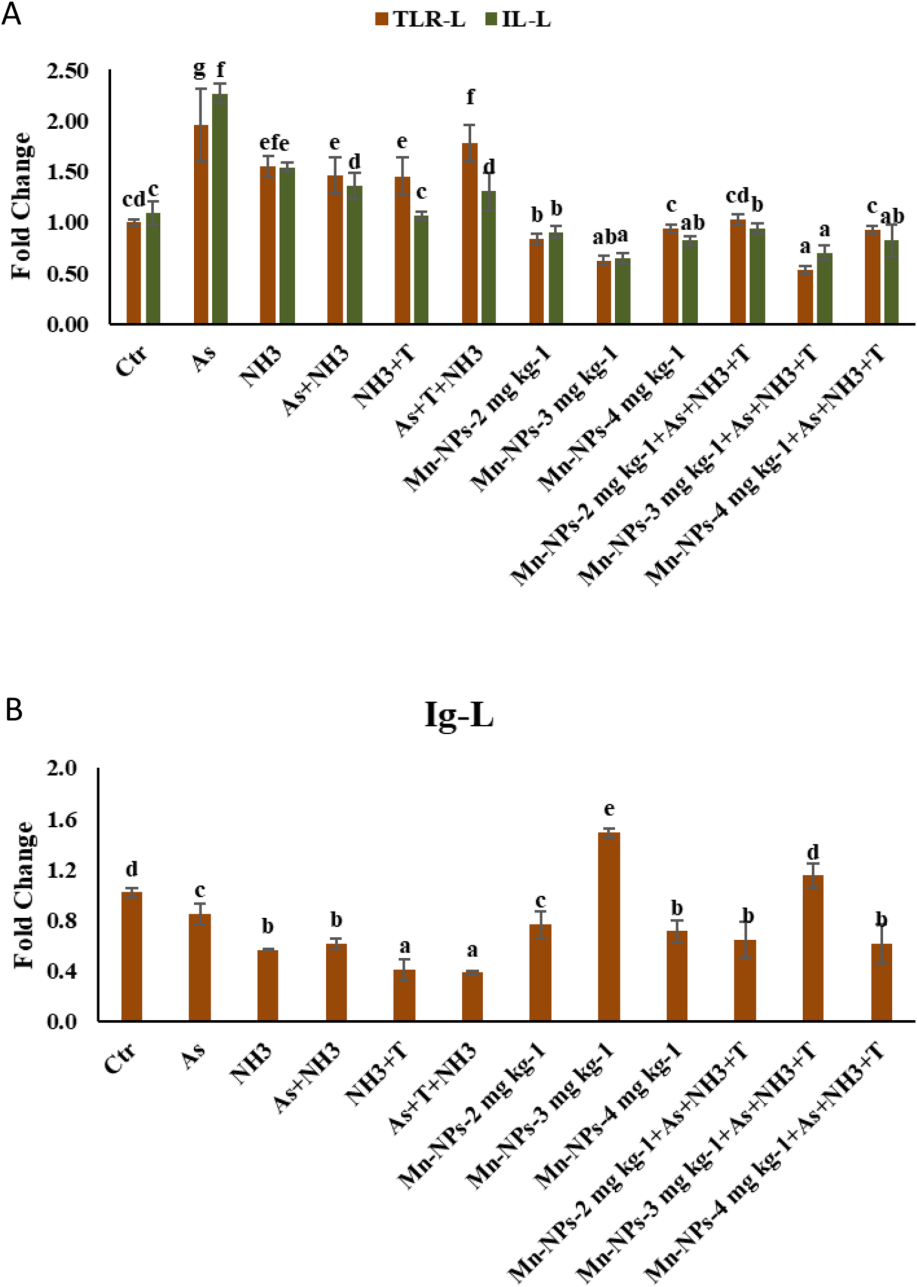
(**A**-**B**): Effect of Mn-NPs on mitigation of aresnic, ammonia and high temperature stress on toll like receptor (TLR), interleukin (IL), and immunglobulin (Ig) gene expressions in fish liver. Within endpoints and groups, bars with different superscripts differ significantly (**a**–**g**). Data expressed as Mean ± SE (n = 3). TLR: (*p* = 0.0038), IL (*p* = 0.0046), Ig (*p* = 0.0042).

Concurrent exposure to arsenic, ammonia and high temperature as well as exposure to arsenic alone, led to a notable upregulation of TLR gene expression (*p* = 0.0038). This upregulation was followed by the NH_3_ + T, As + NH_3_, and NH_3_ groups when compared to both the control and Mn-NPs supplemented groups. Conversely, TLR expression exhibited downregulation in response to Mn-NPs at 3 mg kg^−1^ diet with or without stressors, when compared to the control and other groups. Similarly, the gene expression of IL1b was substantially upregulated (*p* = 0.0046) in response to arsenic exposure, followed by NH_3_, As + NH_3_ + T and As + NH_3_ groups in comparison to the control and Mn-NPs supplemented groups. However, supplementation of Mn-NPs at 3 mg kg^−1^ diet followed 2 mg kg^−1^ diet, resulted in a significantly downregulation (*p* < 0.05) of IL1b expression compared to control and other groups (Fig. 5A). In contrast to the patterns observed for TLR and IL1b, the results of Ig gene expression showed an inverse trend. Notably, the Ig gene expression was significantly (*p* = 0.0042) upregulated upon supplementation of Mn-NPs at 3 mg kg^−1^ diet with or without stressors, compared to the control and other groups. Conversely, exposure to As + NH_3_ + T, NH_3_, As + NH_3_, NH_3_ and As, led to a significant downregulation of Ig gene expression in comparison to the control and other groups (Fig. 5B).

### Secondary stress response (CAS 3a, CAS 3b, CYP450 and MT) with respect to multiple stresses and mitigate it with Mn-NPs

**Figure 6:**
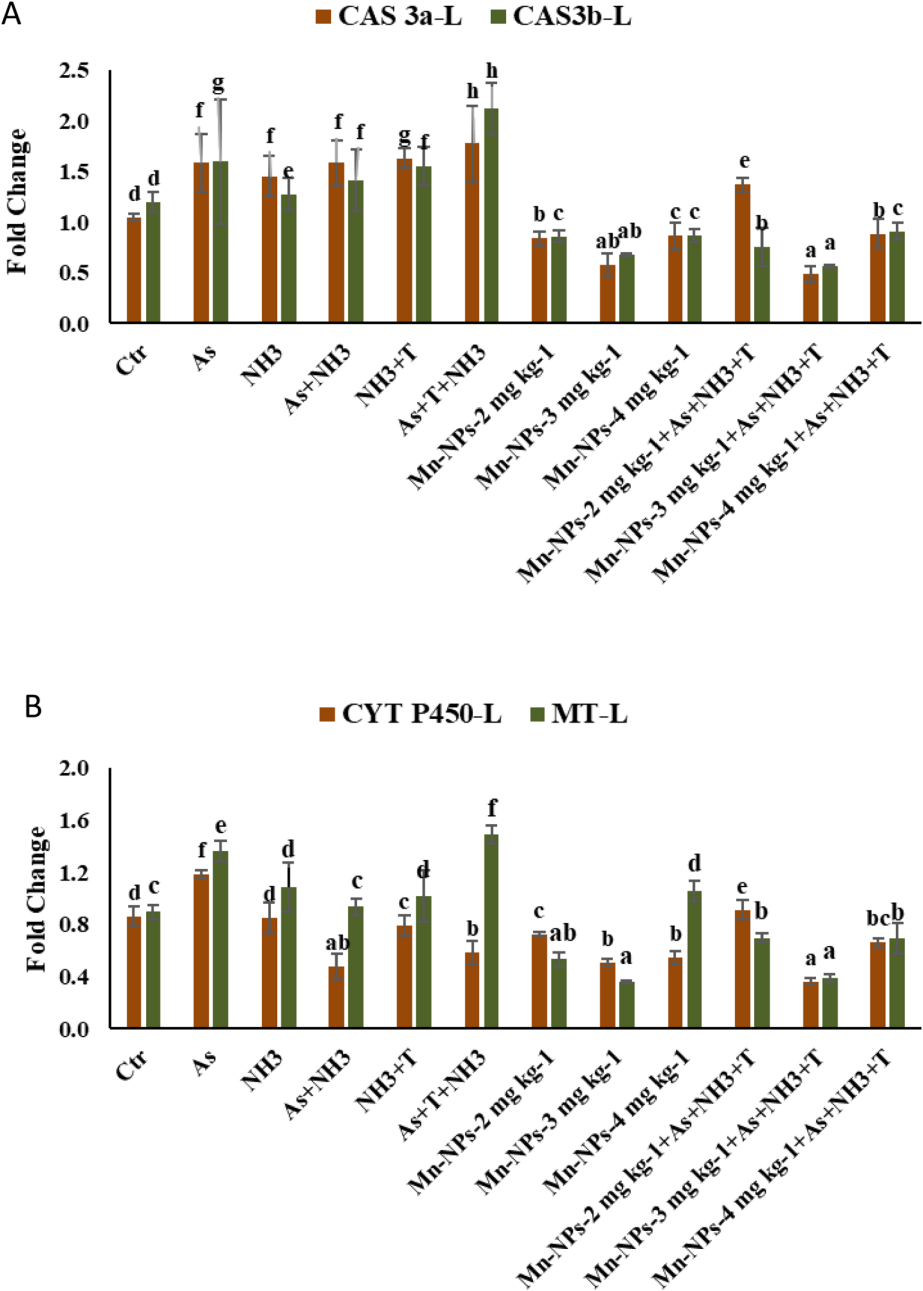
(**A**-B): Effect of Mn-NPs on mitigation of aresnic, ammonia and high temperature stress on caspase (Cas 3a and 3b), cytochrome P450 (CYP P450) and metathionine (MT) gene expressions in fish liver. Within endpoints and groups, bars with different superscripts differ significantly (**a**–**h**). Data expressed as Mean ± SE (n = 3). Caspase 3a: (*p* = 0.0012), Caspase 3b: (*p* = 0.0027), CYP450 (*p* = 0.0031), MT (*p* = 0.0059).

The modulation of gene regulation involving CAS 3a, CAS 3b, CYP450 and MT were investigated in liver tissue of *P. hypophthalmus* reared under As, NH_3_, and high temperature and fed with different level of Mn-NPs and results are presented in Fig. 6A–B. Notably, the expression of CAS 3a (*p* = 0.0012) and CAS 3b (*p* = 0.0027) genes exhibited discernible upregulation when concurrent exposure to As + NH3 + T compared to both control and other treatment groups. Furthermore, the group subjected to exposure to NH_3_ + T, As + NH_3_, NH_3_ and As also demonstrated upregulation of CAS 3a and CAS 3b expression. Conversely, the supplementation of Mn-NPs at 3 mg kg^−1^ diet with or without stressors compared the control and other groups (Fig. 6A). Similarly, the expression of CYP450 exhibited substantial upregulation (*p* = 0.0031) in response to arsenic alone compared to control and other groups. Moreover, the CYP450 was noticeably downregulation with application of Mn-NPs at 3 mg kg^−1^ diet with or without stressors in comparison to control and other groups. Moreover, in case of MT, the expression was noticeably (*p* = 0.0059) upregulated in response to concurrent exposure to As + NH_3_ + T followed by As, NH_3_ + T and NH_3_ groups, in comparison to control and other treatment groups. Conversely, the supplementation of Mn-NPs at 3 mg kg^−1^ diet with or without stressors, led to a significantly downregulation of MT expression in comparison to the control and other supplemented groups (Fig. 6B).

### Secondary stress response (CAT, SOD, GPx and Na^+^K^+^ATPase) with respect to multiple stresses and mitigate it with Mn-NPs

**Figure 7:**
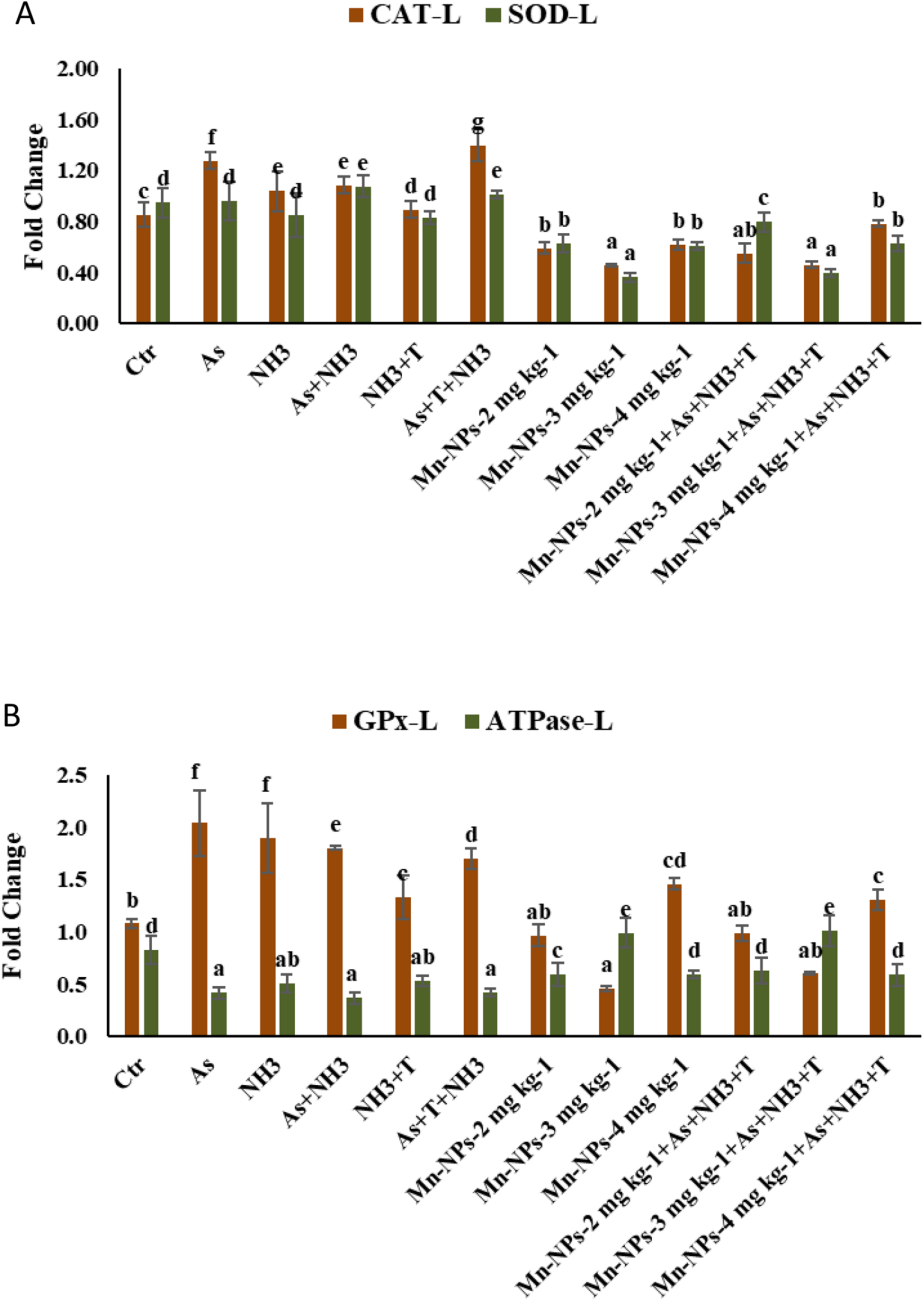
(**A**-**B**): Effect of Mn-NPs on mitigation of aresnic, ammonia and high temperature stress on catalase (CAT), superoxide dismutase (SOD), glutathion peroxidase (GPx) and Na^+^K^+^ ATPase gene expressions in fish liver. Within endpoints and groups, bars with different superscripts differ significantly (**a**–**g**). Data expressed as Mean ± SE (n = 3). CAT (*p* = 0.0026), SOD (*p* = 0.012), GPx (*p* = 0.017) and Na^+^K^+^ ATPase (*p* = 0.0011).

The expressions of CAT (*p* = 0.0026) and SOD (*p* = 0.012) genes demonstrated substantial and noteworthy upregulation upon concurrent exposure to As + NH3 + T, in comparison to both the control and the supplemented groups. Conversely, Mn-NPs at 3 mg kg^−1^ diet followed by 2 and 4 mg kg^−1^ diet groups with or without stressors, led to noticeable downregulation of CAT and SOD gene expression compared to control and stressors groups (Fig. 7A). Furthermore, the gene expression of GPx in liver tissue exhibited a noticeable upregulation in response to As, NH_3_, As + NH_3_ and As + NH_3_ + T compared to control and supplemented groups. Whereas, Mn-NPs at 3 mg kg^−1^ diet led to a significant downregulation of GPx (*p* = 0.017) gene expression. Remarkably, the result of Na^+^K^+^ATPase (*p* = 0.0011) gene expression displayed an inverse pattern in relation to GPx (Fig. 7B).

### Tertiary stress response (GH, MYST, SMT, GHR1, GHRβ, IGF1, IGF2) with respect to multiple stresses and mitigate it with Mn-NPs

**Figure 8:**
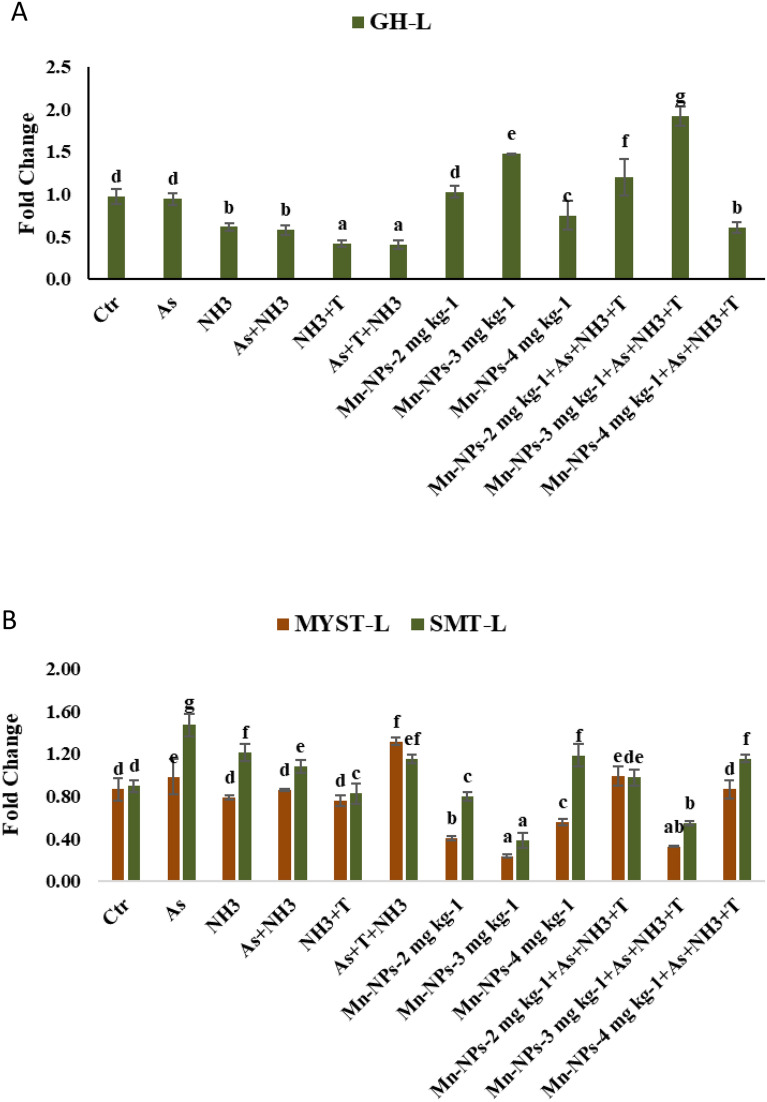
(**A**-**B**): Effect of Mn-NPs on mitigation of aresnic, ammonia and high temperature stress on growth hormone (GH) in liver, myostatin (MYST) and somatostatin (SMT) gene expressions in fish liver. Within endpoints and groups, bars with different superscripts differ significantly (**a**–**g**). Data expressed as Mean ± SE (n = 3). GH (*p* = 0.0013) MYST (*p* = 0.0047) and SMT (*p* = 0.0088).

**Figure 9:**
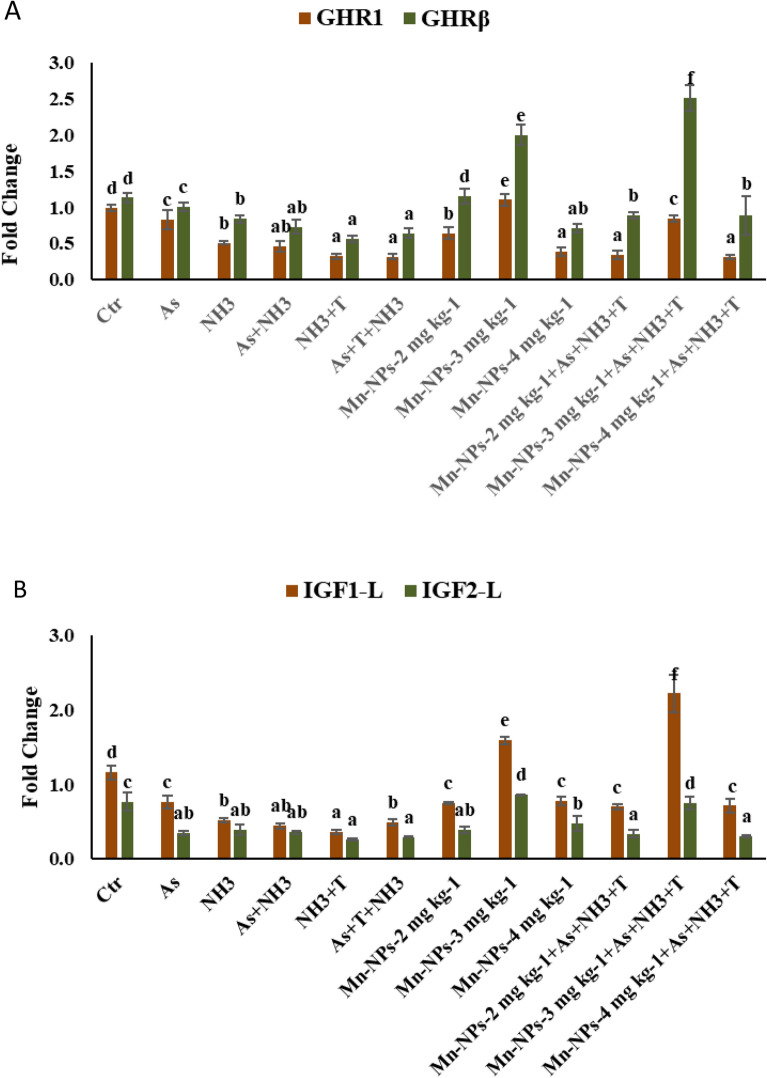
(**A**-**B**): Effect of Mn-NPs on mitigation of aresnic, ammonia and high temperature stress on growth hormone regulator 1 and β (GHR1 and GHRβ), insuline like growth factor 1 and 2 in (IGF1 and IGF 2) gene expressions in liver tissue of fish. Within endpoints and groups, bars with different superscripts differ significantly (**a**–**f**). Data expressed as Mean ± SE (n = 3). GHR1 (*p* = 0.0014), GHRβ (*p* = 0.0029), IGF1 (*p* = 0.0017) and IGF 2 (*p* = 0.014).

In the present investigation, the gene expression of GH was substantially downregulated (*p* = 0.0013) in the group exposed to As + NH_3_ + T, NH_3_ + T followed by As + NH_3_ and NH_3_ groups. However, when supplemented with Mn-NPs at 3 mg kg^−1^ diet with or without stressors (As + NH_3_ + T), GH expression was significantly upregulated compared to the control and other groups (Fig. 8A). Furthermore, MYST expression was notably upregulated (*p* = 0.0047) following exposure to As + NH_3_ + T, followed by As, while SMT expression was highly upregulated (*p* = 0.0088) in the As followed by As + NH_3_ + T group, in comparison to the control and Mn-NPs at 2 and 3 mg kg^–1^ diet groups. In contrast, both MYST and SMT expression were remarkably downregulated with Mn-NPs at 3 mg kg^-1^, followed by 2 mg kg^−1^ diet, with or without stressors. The results from the present study showed that the supplemented group with Mn-NPs at 4 mg kg^-1^ diet was not effective in regulating the MYST and SMT expression in the liver tissue of *P. hypophthalmus* (Fig. 8B). Interestingly, GHR1 (*p* = 0.0014) and GHRβ (*p* = 0.0029) expressions were noticeably downregulated with exposure to As + NH_3_ + T, NH_3_ + T, As + NH_3_ followed by NH_3_ and As groups, in comparison to the control and other groups. However, the gene expression of GHR1, and GHRβ were significantly upregulated with Mn-NPs at 3 mg kg^−1^ diet, compared to control and other groups. The supplemented groups with Mn-NPs at 2 and 4 mg kg^−1^ diet were not effective to modulate the multiple stresses (Fig. 9A). Similarly, IGF1 and IGF2 expression were determined in the liver tissues of *P. hypophthalmus* exposed to arsenic, ammonia and high temperature stress (Fig. 9B). Both IGF1 (*p* = 0.0017) and IGF2 (*p* = 0.014) expressions were noticeably downregulated with exposure to As + T + NH_3_, NH_3_ + T, As + NH_3_, NH_3_ and As groups, compared to the control and supplemented group (Mn-NPs at 3 mg kg^−1^ diet). Interestingly, IGF1 expression was significantly highly upregulated with Mn-NPs at 3 mg kg^−1^ diet in comparison to control and other groups.

### Tertiary stress response (final body weight gain %, FCR, SGR, PER, DGI %, TGC and RFI) with respect to multiple stresses and mitigate it with Mn-NPs

The growth performance of *P. hypophthalmus* reared under multiple stresses (As + NH_3_ + T, NH_3_ + T, As + NH_3_, NH_3_, As) was assessed in the present study, and the corresponding data are documented in Table 5. The final body weight gain % (*p* = 0.0055), SGR (*p* = 0.0027), PER (*p* = 0.0032), DGI (*p* = 0.017) and RFI (*p* = 0.013) were significantly reduced in presence of stressors (As + NH_3_ + T, NH_3_ + T, As + NH_3_, NH_3_, As) when compared to both the control and Mn-NPs supplemented groups. Interestingly, the supplementation of Mn-NPs at 3 mg kg^−1^ diet, followed by Mn-NPs at 2 mg kg^−1^ diet, remarkably enhanced the indicators of body weight gain %, SGR, PER, DGI and RFI. In contrast to results of body weight gain %, the FCR exhibited a significant (*p* = 0.0043) inverse relationship with other growth performance metrics. Notably, Mn-NPs at 3 mg kg^−1^ diet demonstrated a superior FCR value compared to other feeds. Furthermore, the TGC did not exhibit significant variation among the treatment groups.

**Table 5:** Mitigating role of Mn-NPs on growth performance (final body weight gain %, FCR, SGR, PER, DGI, TGC and RFI) of *P. hypophthalmus* reared under arsenic, ammonia and high temperature stress for 105 days.

Treatments	Final body weight gain %	FCR	SGR	PER	DGI (%)	TGC	RFI
Control	102.63f. ± 0.42	3.10^c^ ± 0.01	0.70^d^ ± 0.01	0.93c ± 0.02	1.06^e^ ± 0.01	0.0396 ± 0.0001	317.65^d^ ± 0.78
As	61.80^ab^ ± 4.50	4.58f. ± 0.31	0.49^ab^ ± 0.01	0.63a ± 0.04	0.68^a^ ± 0.04	0.0404 ± 0.0002	280.04^a^ ± 0.65
NH_3_	68.43^c^ ± 0.60	4.13^e^ ± 0.03	0.49^ab^ ± 0.02	0.69a ± 0.03	0.75^b^ ± 0.03	0.0389 ± 0.0001	282.36^a^ ± 0.31
As + NH_3_	64.69^b^ ± 1.84	4.39^e^ ± 0.11	0.50^b^ ± 0.01	0.67a ± 0.02	0.71^a^ ± 0.02	0.0390 ± 0.0002	283.90^a^ ± 1.14
NH_3_ + T	62.42^ab^ ± 3.30	4.54f. ± 0.22	0.49^ab^ ± 0.03	0.69a ± 0.04	0.69^a^ ± 0.03	0.0308 ± 0.0001	281.77^a^ ± 0.99
As + T + NH_3_	59.51^a^ ± 4.01	4.70^ g^ ± 0.28	0.46^a^ ± 0.02	0.67a ± 0.05	0.66^a^ ± 0.04	0.0306 ± 0.0001	277.58^a^ ± 1.15
Mn-NPs-2 mg kg^-1^	130.10^ g^ ± 7.54	2.55^b^ ± 0.14	0.75^e^ ± 0.03	1.23e ± 0.08	1.29f. ± 0.06	0.0391 ± 0.0002	329.29^e^ ± 2.44
Mn-NPs-3 mg kg^-1^	196.74^ h^ ± 3.33	1.94^a^ ± 0.02	1.04f. ± 0.01	1.67f. ± 0.02	1.78^ g^ ± 0.02	0.0382 ± 0.0001	381.79f. ± 3.79
Mn-NPs-4 mg kg^-1^	74.53^d^ ± 2.53	3.95^d^ ± 0.11	0.57^c^ ± 0.02	0.73b ± 0.03	0.81^c^ ± 0.02	0.0400 ± 0.0001	293.71^b^ ± 2.07
Mn-NPs-2 mg kg^-1^ + As + T + NH_3_	125.45^ g^ ± 1.06	2.62^b^ ± 0.01	0.76^e^ ± 0.03	1.12d ± 0.01	1.26f. ± 0.01	0.0305 ± 0.0002	328.08^e^ ± 2.42
Mn-NPs-3 mg kg^-1^ + As + T + NH_3_	206.72^i^ ± 3.90	1.85^a^ ± 0.03	1.06f. ± 0.01	1.59f. ± 0.07	1.88^ g^ ± 0.03	0.0308 ± 0.0001	381.55f. ± 2.73
Mn-NPs-4 mg kg^-1^ + As + T + NH_3_	89.32^e^ ± 2.20	3.41^d^ ± 0.04	0.66^d^ ± 0.04	0.93c ± 0.03	0.95^d^ ± 0.02	0.0308 ± 0.0002	304.68^c^ ± 4.30
*P*-value	0.0055	0.0043	0.0027	0.0032	0.017	NS	0.013

Values in the same row with different superscript (a, b, c, d, e) differ significantly. Data expressed as Mean ± SE (n = 3). FCR: feed conversion ratio; SGR: specific growth rate; PER: protein efficiency ratio; DGI: Daily growth index; TGC: Thermal growth coefficient; RFI: relative feed intake.

### Tertiary stress response (Bioaccumulation of arsenic) with respect to multiple stresses and mitigate it with Mn-NPs

**Figure 10:**
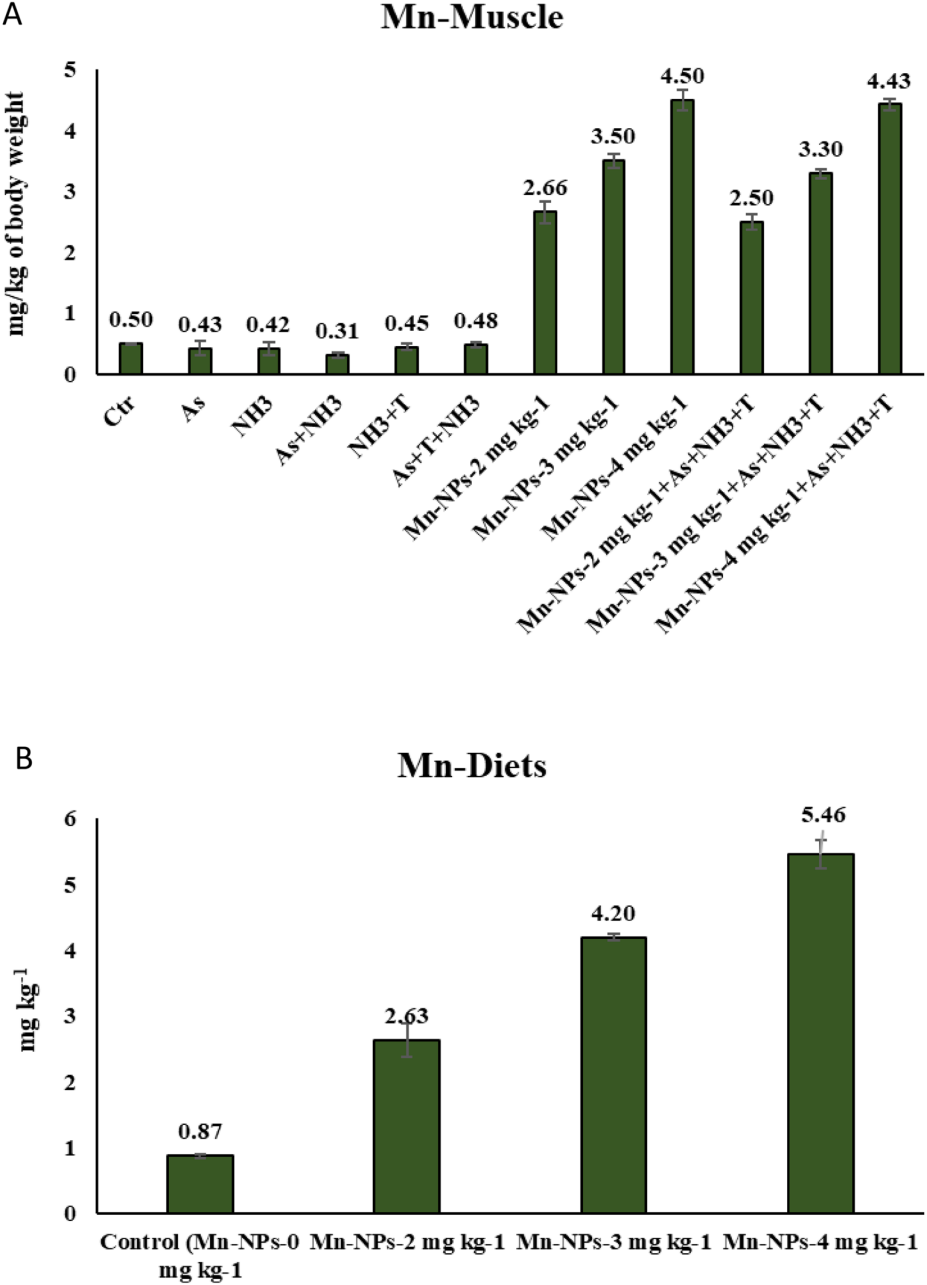
(**A**-**B**): Manganese concentrations in muscle and diets.

The analysis of arsenic bioaccumulation was conducted across various fish tissues, including the liver, kidney, muscle, brain and gill. The concentration of arsenic in the experimental water was measured, and the findings are presented in Table 6. Among the tested groups, the highest arsenic concentration was observed in the As + NH_3_ + T (2140 µg L^−1^), As + NH_3_ (1780 µg L^−1^) and As (1462 µg L^−1^) treatments groups. Notably, the supplemented groups of Mn-NPs at 3, 2 and 4 mg kg^−1^ diet with stressors exhibited arsenic concentration of 685 µg L^−1^, 1207 µg L^−1^, 1472 µg L^−1^ respectively. Further examination of arsenic bioaccumulation revealed the highest levels in the kidney tissue (13.60 mg kg^−1^ tissue) of the group exposed to As + NH_3_ + T, followed by liver (12.43 mg kg^−1^ tissue) and gill (8.68 mg kg^−1^ tissue). Remarkably, the inclusion of Mn-NPs at 3 mg kg^−1^ diet substantially reduced arsenic bioaccumulation across all the tissues. In fact, arsenic was undetectable in the muscle and brain tissues of the group treated with Mn-NPs at 3 mg kg^−1^ diet. Additionally, the manganese content in both muscle tissue and fish diets was analysed and is presented in Fig. 10A–B. The highest bioaccumulation of Mn occurred in the group treated with Mn-NPs at 4 mg kg^−1^ diet, with (4.43 mg kg^−1^) and without stressors (4.5 mg kg^−1^ diet). Similarly, Mn content in diets was recorded as 0.87, 2.63, 4.20 and 5.46 mg kg^−1^ diet in the control and Mn-NPs at 2, 3 and 4 mg kg^−1^ diets respectively.

**Table 6:** Effect of Mn-NPs on arsenic bioaccumulation in water and different fish tissues of *P. hypophthalmus* reared under arsenic, ammonia and high temperature stress for 105 days.

Treatments	Water (µg L^−1^)	Liver	Muscle	Gill	Kidney	Brain
Control	0.32 ± 0.04	0.17 ± 0.03	ND	0.13 ± 0.02	0.30 ± 0.01	ND
As	1462.69 ± 44.17	7.95 ± 0.54	0.33 ± 0.06	4.44 ± 0.43	8.95 ± 0.31	0.12 ± 0.0
NH_3_	0.55 ± 0.08	0.19 ± 0.01	0.11 ± 0.02	0.87 ± 0.02	0.68 ± 0.002	0.10 ± 0.001
As + NH_3_	1780.95 ± 5.75	11.12 ± 0.55	0.82 ± 0.05	7.35 ± 0.11	11.82 ± 0.41	0.13 ± 0.01
NH_3_ + T	0.04 ± 0.01	0.14 ± 0.02	ND	0.04 ± 0.01	0.09 ± 0.01	ND
As + T + NH_3_	2140.37 ± 72.23	12.43 ± 0.23	1.20 ± 0.02	8.68 ± 0.38	13.60 ± 0.49	0.11 ± 0.01
Mn-NPs-2 mg kg^−1^	0.27 ± 0.03	0.16 ± 0.04	ND	0.06 ± 0.01	0.07 ± 0.01	ND
Mn-NPs-3 mg kg^−1^	0.30 ± 0.01	0.03 ± 0.0	ND	0.06 ± 0.02	0.02 ± 0.0	ND
Mn-NPs-4 mg kg^−1^	0.34 ± 0.02	0.15 ± 0.02	ND	0.17 ± 0.01	0.76 ± 0.03	ND
Mn-NPs-2 mg kg^−1^ + As + T + NH_3_	1207.56 ± 29.33	10.42 ± 0.56	0.42 ± 0.10	4.67 ± 0.50	14.09 ± 0.14	0.88 ± 0.03
Mn-NPs-3 mg kg^−1^ + As + T + NH_3_	685.07 ± 21.92	5.40 ± 0.35	0.62 ± 0.11	2.18 ± 0.41	7.29 ± 0.21	0.03 ± 0.0
Mn-NPs-4 mg kg^−1^ + As + T + NH_3_	1472.99 ± 27.28	13.82 ± 0.03	1.48 ± 0.21	7.95 ± 0.61	15.14 ± 1.10	1.02 ± 0.02

Data expressed as Mean ± SE (n = 3). ND: Not detectable.

## Discussion

The present study, investigates the role of Mn-NPs in mitigating the impact of multiple stresses such as arsenic and ammonia toxicity, along with high temperature stress in *P. hypophthalmus*. This study represents the first of its kind to elucidate the mechanistic role of Mn-NPs in alleviating these multifaceted stresses by regulating several genes involved in stress response. The study delves into primary, secondary and tertiary stress responses, detailing the ways in which Mn-NPs can effectively address these challenges. Specifically, the primary stress response, characterised by cortisol secretion induced by arsenic and ammonia toxicity and high temperature stress was examined. The heightened cortisol levels resulting from ammonia, arsenic and high temperature stress could be attributed to the excessive release of catecholamines during gluconeogenesis and glycogenolysis in fish, brought about by stressors like As + NH_3_ + T^32^. The cortisol surge assumes a crucial role in providing energy to lipid, glucose and fatty acid during stress, thereby maintaining homeostasis^33^. It also ensures an adequate energy supply to match cellular demands during stress condition^34^ while concurrently stimulating the release of ACTH (adrenocorticotrophic hormone) from anterior pituitary gland^35^. The stressors (As + NH_3_ + T, NH_3_ + T, As + NH_3_, NH_3_, As) could potentially impact various points along the hypothalamus-pituitary-interrenal axis, disrupting ACTH secretion^36,37^. Elevated cortisol expression might negatively effect on health status of the fish, considering its involvement in immunity, osmoregulation and metabolism^36^. Strikingly, this study is the first to explore the role of Mn-NPs in cortisol reduction, which contributes to stress alleviation. Notably, Mn-NPs assume a significant role in regulation of blood glucose regulation, counteracting reactive oxygen species (ROS), maintaining nervous system functionality, aiding blood coagulation, serving as an immune-modulator and promoting bone growth^13,14^. Mn-NPs participated in the biochemical reactions as integral components of metalloenzymes and as enzymes activators. This plausible mechanism could underlie Mn-NPs capacity to lower cortisol levels during stress conditions (As + NH_3_ + T, NH_3_ + T, As + NH_3_, NH_3_, As). Remarkably, Mn-NPs at 3 mg kg^−1^ diet followed by 2 mg kg^−1^ diet, substantially reduced cortisol levels. Furthermore, alongside cortisol, Mn-NPs at 3 mg kg^−1^ diet also demonstrated the ability to regulate the expression of HSP 70. The highly conserved chaperone protein plays a pivotal role in processes such as protein synthesis, folding, aggregation, prevention, transport and translocalization of protein^38^. Indeed, the Mn-NPs at 3 mg kg^−1^ diet have controlled the regulation of HSP 70 gene expression. This could be due to role of Mn in activating the transcription factor of HSP which lose the binding activity of heat shock elements and thus they might be downregulated the HSP 70 expression in liver tissue of *P. hypophthalmus*^39,40^.

The present investigation has also unveiled that the inclusion of Mn-NPs at 3 mg kg^−1^ serves as a safeguard against DNA damage and enhances the expression of DNA damage inducible protein (DDIP) in fish subjected to various stressors. This positive outcome could potentially be attributed to the upregulation of SOD gene expressions and 8-hydroxydeoxyguanosine (8-OHdG), which is indicative of DNA damage. Notably, this study demonstrated the modulation of SOD by stressors, particularly, in the context of (As + NH_3_ + T)^12^. In this line of investigation, the induction of the iNOS gene expression resulting from ammonia toxicity, arsenic pollution and high temperature stress was observed. This induction could be linked to the elevated accumulation of NH_3_ in fish tissues. Conventionally, toxic NH_3_ undergoes conversion into urea through the ornithine-urea cycle (OUC) and transform into glutamine, incorporating non-essential amino acids^41^. Interestingly, nitric oxide (NO) contributes to shielding cellular systems against oxidative stress^42^. The results showed that higher expression of iNOS in liver tissues probably due to substantial presence of NH_3_ in the bloodstream^43^. Reduced feed intake, stemming from heightened NH_3_ bioaccumulation in tissues, potentially triggers the upregulation of the iNOS gene expression. Surprisingly, the administration of Mn-NPs at 3 mg kg^−1^ diet was associated with a discernible downregulation, effectively controlling the regulatory mechanisms governing this gene.

In the course of this investigation, the combination of ammonia toxicity, arsenic pollution and high temperature stress exerted notable effects on the expressions of immune related genes such as TLR, IL, TNFα and Ig. Intriguingly, the incorporation of dietary Mn-NPs exhibited a capacity to bolster the immune response in fish exposed stressors (As + NH_3_ + T, NH_3_ + T, As + NH_3_, NH_3_ and As groups). The study conducted by Zhang et al. revealed that the ammonia toxicity altered the immunity of the fish. Pro-inflammatory cytokines like TNFα, IL and TLR plays a pivotal role in enhancing immunity in aquatic animals including fish^44–46^. The stressors showed that higher inflammation rate in liver tissue in fish and hence the higher upregulation of TNFα, IL and TLR was determined in the present study^9^. Surprisingly, the introduction of Mn-NPs at 3 mg kg^−1^ diet demonstrated a dampening effect on the inflammatory response in liver tissues, achieving a downregulation of the gene expression associated with TNFα, IL, and TLR. This intriguing outcome could potentially be attributed to the essential immunostimulant properties of Mn-NPs, which likely activate the NF-κB signaling pathway, thus reinforcing the fish's immune defense against multifaceted stressors. Furthermore, Mn-NPs exhibited the ability to upregulate the gene expression of immunoglobulin (Ig), thus contributing to enhanced immune activity in fish. Conversely, the stressors (As + NH_3_ + T, NH_3_ + T, As + NH_3_, NH_3_, and As groups) induced a reduction in Ig expression, thereby compromising immunity through the suppression of humoral and cell-mediated responses. Mn-NPs may offer a multifaceted contribution to immunity enhancement, potentially encompassing improved antibody affinity, early B cell development, reinforcement of the complement system, fortification of cell-mediated immunity, heightened phagocytosis activity, and potentiated antibody reactions.

In the present investigation, exposure to multiple stressors in fish elicited the induction of Cas 3a and Cas 3b. It is an important component of apoptosis which could be related with oxidative stress and inflammatory response^47^. Notably, the influence of ammonia, arsenic and high temperature stress on fish physiology could potentially trigger apoptosis, orchestrated through the action of p53, a pivotal regulator in apoptosis pathways^48^. These findings suggest that CYP 450 may play a role in apoptosis induced by multiple stressors, potentially through the upregulation of the transcription of bcl2-associated X (Bax)^49^. Surprisingly, Bax, a significant contributor to cell death in fish, initiates the release of cytochrome c, ultimately activating caspases^50^. Remarkably, dietary supplementation of Mn-NPs at 3 mg kg^−1^ diet exhibited a protective effect against apoptosis, as evidenced by the downregulation of Cas 3a and Cas 3b gene expressions. This protective mechanism could be attributed to manganese's pivotal role as a constituent of MnSOD, effectively scavenging superoxide radicals^51^. Located within the mitochondria, MnSOD aids in reducing O_2_- levels, consequently assuming a neuroprotective role.

In the present study, the expression of the CYP450 was observed to be upregulated in response to ammonia toxicity, arsenic pollution and high temperature. This gene is integral to the metabolic pathway involving arachidonic acid, lipoxygenase and cyclooxygenase pathways. Notably, CYP450 epoxygenases facilitate the transformation of arachidonic acid, leading to the production of various regioisomers of EETs (14,15-epoxyeicosatrienoic acids)^52^. Interestingly, the inclusion of dietary Mn-NPs yielded a contrasting effect by downregulating the expression of the CYP450 gene, subsequently alleviating the impact of multiple stressors. This phenomenon could potentially be attributed to the utilization of manganese by certain CYP450 and SOD enzymes in their catalytic activities, potentially replacing the role of iron. Similarly, the gene expression of metallothionein (MT), a protein involved in metal detoxification and homeostasis, exhibited an overexpression in response to ammonia, arsenic, and high-temperature stress, which was subsequently mitigated by the Mn-NPs diet. Furthermore, the augmented intake of dietary Mn-NPs contributed to the overexpression of the MT gene^53^.

Dietary supplementation of Mn-NPs yielded enhancements in the gene expression of SOD, CAT, GPx and ATPase. On a related note, the present investigation revealed that arsenic and ammonia contamination, coupled with high-temperature stress, induced alterations in the expression of anti-oxidative and ATPase genes. Interestingly, manganese emerged as a protective element, shielding fish from oxidative stress brought about by a multitude of stressors. This protective role could be attributed to manganese’s multifaceted involvement, serving as a cofactor for several enzymes, including pyruvate carboxylase and manganese superoxide dismutase (Mn-SOD). Furthermore, manganese stands as a vital component within metalloenzymes, acting as a defender against reactive oxygen species (ROS) by catalyzing the one-electron reduction of peroxide anion to hydrogen peroxide^54^. The observed downregulation of CAT, SOD, and GPx could conceivably align with the aforementioned mechanism, acting as a protective response against the array of stresses present (As + NH_3_ + T). Significantly, CAT and SOD function as the frontline defense mechanisms against oxidative stress by catalyzing the dismutation of O_2_ into H_2_O_2_ and O_2_ within the cellular milieu. It is noteworthy that Mn-SOD plays a pivotal role in maintaining the structural integrity of antioxidant enzymes^55,56^. Furthermore, CAT, localized within peroxisomes, aids in counteracting oxidative stress by efficiently converting H_2_O_2_ into H_2_O and O_2_^57^. Additionally, GPx assumes a crucial role in detoxifying H_2_O_2_, an action facilitated by SOD, thereby contributing to the preservation of cellular homeostasis.

Fascinatingly, the dietary supplementation of Mn-NPs exhibited an upregulation in the growth-related gene, encompassing GHR1, GHRβ, GH, IGF1, IGF2 while concurrently downregulating the expression of SMT and MYST. In contrast, the presence of stressors such as arsenic and ammonia toxicity and high temperature induced noteworthy shifts in the expression patterns of growth-related genes, ultimately resulting in diminished growth performance metrics encompassing body weight gain %, SGR, PER, FCR, RFI and daily growth index. These effects may be attributed to the interplay between arsenic, ammonia toxicity, and high temperature stress, culminating in reduced feed intake and metabolic rates, a phenomenon previously documented in our earlier study^58^. Surprisingly, the inclusion of dietary Mn-NPs diets exerted a remarkable positive influence, substantially enhancing feed efficiency, feed utilization, growth rate, and immunity of the fish. Furthermore, Mn-NPs enriched diets yielded improvement in specific growth rate, daily growth index %, relative feed intake and protein efficiency in the fish^59^. The pivotal growth hormone gene (GH), functioning in concert with its receptor GHR, assume a crucial role in growth and developmental processes. The regulation of GH is controlled by hypothalamic regulation viz. GH-releasing hormone, ghrelin, dopamine and somatostatin^60,61^. While growth is inherently modulated by genetic, endocrinological, and environmental factors, optimal growth performance is often attributed to a harmonious blend of balanced nutrition, favorable temperature conditions, optimal husbandry practices, and finely tuned endocrine regulation^62^. Consequently, within the context of the present study, Mn-NPs were shown to significantly enhance growth performance by modulating the expression of key growth-performing genes. Interestingly, the expression of MYST and SMT genes were noticeably upregulated in response to stressors, whereas dietary supplementation of dietary Mn-NPs at 3 mg kg^−1^ diet effectively downregulated the expression of both SMT and MYST genes. MYST, in particular, is known to impede myoblast function, leading to terminal differentiation and fiber enlargement^63^. Conversely, the dynamic regulation of IGF-1 and IGF-2 in liver cells was impacted by ammonia and arsenic toxicity, alongside high temperature stress, with Mn-NPs intervention leading to an augmentation in their regulatory functions. Notably, GH’s interaction with its receptor in liver tissues triggers the release and synthesis of IGF-1, which, in turn, participates in pivotal biomolecular regulations, including carbohydrate, lipid, protein, and mineral metabolism, as well as cellular differentiation and proliferation, culminating in growth^64^. Remarkably, the bioaccumulation of arsenic within distinct fish tissues such as muscle and brain tissues were rendered undetectable in the Mn-NPs supplemented groups including the control and non-arsenic-exposed groups. Among the groups concurrently exposed to arsenic, ammonia, and high temperature stress, the cohort supplemented with Mn-NPs at 3 mg kg^−1^ diet exhibited the lowest arsenic bioaccumulation across all tissues, notably outperforming control and other treatment groups. This phenomenon can be attributed to manganese's adeptness in enhancing arsenic detoxification across various tissues. Notably, higher bioaccumulation of arsenic was observed in kidney and liver tissues. Collectively, these results underscore the potent detoxification capabilities of Mn-NPs, efficiently mitigating arsenic's impact across diverse tissue types.

## Conclusion

The current study focuses on the impact of Mn-NP supplementation on gene regulation related to various aspects of growth performance, immunity, antioxidative capacity, genotoxicity, and stress responses in *P. hypophthalmus*. Specifically, the study investigates how Mn-NPs, administered at a concentration of 3 mg kg^−1^ in the diet, influence the expression of genes such as cortisol, HSP 70, apoptosis-related genes, and those associated with genotoxicity. Furthermore, the study explores the protective effects of Mn-NPs against the stresses induced by arsenic and ammonia pollution, as well as high temperature. The findings demonstrate that the presence of Mn-NPs leads to the positive modulation of gene expression related to cortisol regulation, HSP 70 production, apoptosis inhibition, and genotoxicity prevention. Notably, the study reveals that the gene regulation pertaining to growth performance is also influenced and enhanced by Mn-NPs, ultimately contributing to improved growth rates in fish exposed to stressful conditions. Remarkably, the administration of Mn-NPs at 3 mg kg^−1^ effectively promotes the detoxification of arsenic across various fish tissues. This investigation is pioneering in its elucidation of the mechanistic role played by Mn-NPs in mitigating the adverse effects of ammonia and arsenic toxicity, as well as high temperature stress, in fish. Importantly, these findings highlight the potential of Mn-NPs at 3 mg kg^−1^ in enhancing the well-being of fish species in the face of the contemporary challenges posed by climate change and pollution.

## Data Availability

The datasets generated during and/or analysed during the current study are available from the corresponding author on reasonable request.
